# Herpes simplex virus type-1 cVAC formation in neuronal cells is mediated by dynein motor function and glycoprotein retrieval from the plasma membrane

**DOI:** 10.1128/jvi.00713-24

**Published:** 2024-06-20

**Authors:** Shaowen White, Richard Roller

**Affiliations:** 1 Department of Microbiology and Immunology, Carver College of Medicine, University of Iowa, Iowa City, Iowa, USA; The University of Arizona, Tucson, Arizona, USA

**Keywords:** herpes simplex, envelopment, virion assembly, dynein, dynactin, plasma membrane retrieval

## Abstract

Herpesvirus assembly requires the cytoplasmic association of large macromolecular and membrane structures that derive from both the nucleus and cytoplasmic membrane systems. Results from the study of human cytomegalovirus (HCMV) in cells where it organizes a perinuclear cytoplasmic virus assembly compartment (cVAC) show a clear requirement for the minus-end-directed microtubule motor, dynein, for virus assembly. In contrast, the assembly of herpes simplex virus -1 (HSV-1) in epithelial cells where it forms multiple dispersed, peripheral assembly sites is only mildly inhibited by the microtubule-depolymerizing agent, nocodazole. Here, we make use of a neuronal cell line system in which HSV-1 forms a single cVAC and show that dynein and its co-factor dynactin localize to the cVAC, and dynactin is associated with membranes that contain the virion tegument protein pUL11. We also show that the virus membrane-associated structural proteins pUL51 and the viral envelope glycoprotein gE arrive at the cVAC by different routes. Specifically, gE arrives at the cVAC after retrieval from the plasma membrane, suggesting the need for an intact retrograde transport system. Finally, we demonstrate that inhibition of dynactin function profoundly inhibits cVAC formation and virus production during the cytoplasmic assembly phase of infection.

**IMPORTANCE:**

Many viruses reorganize cytoplasmic membrane systems and macromolecular transport systems to promote the production of progeny virions. Clarifying the mechanisms by which they accomplish this may reveal novel therapeutic strategies and illustrate mechanisms that are critical for normal cellular organization. Here, we explore the mechanism by which HSV-1 moves macromolecular and membrane cargo to generate a virus assembly compartment in the infected cell. We find that the virus makes use of a well-characterized, microtubule-based transport system that is stabilized against drugs that disrupt microtubules.

## INTRODUCTION

Herpesvirus infection requires the movement of large macromolecular complexes and membrane vesicles in the cytoplasm of the host cell. For example, capsids must be moved at the initiation of viral infection from the cell periphery, where the incoming virus envelope fuses with the host plasma membrane, to the nuclear envelope, where the viral genome is injected into the nucleus. Later in infection, after the nucleocapsid has egressed from the nucleus, the capsid and vesicles that carry membrane-associated virus structural proteins must arrive at the site of cytoplasmic envelopment. DNA-containing herpesvirus capsids are ~125 nm in diameter, and measurements of the movement of injected nanoparticles of this size in the cytoplasm of a variety of cell types suggest that a nucleocapsid would be unable to move by diffusion in this environment (1). The membrane compartments into which the nucleocapsid buds during cytoplasmic envelopment must be even larger to contain the mature enveloped virion after it has formed. These theoretical considerations strongly suggest that both virus entry, assembly, and post-assembly transport must require molecular motors.

Dynein is a multi-subunit complex that mainly serves as a microtubule-associated motor but can also function to promote clustering of microtubule minus ends at the microtubule organization center (MTOC) (2) or to connect microtubule plus ends to the cell periphery (3, 4). The motor function of dynein requires a cargo adapter and processivity factor called dynactin, which is a 23-subunit protein complex (5, 6). The largest of these subunits is p150Glued which interacts both with cargo molecules and with dynein itself by way of the dynein intermediate chain (DIC). The interaction between p150Glued and DIC is mediated in part by the first coiled-coil domain (CC1) of p150Glued, and the overexpression of the CC1 domain alone interferes with that interaction and inhibits dynein motor function (7).

Alphaherpesvirus capsid and virion movement over long distances require microtubules and microtubule-associated motors. Initiation of infection by HSV and pseudorabiesvirus (PrV) in differentiated neurons requires the engagement of dynein motors by the virion tegument component pUL36/VP1/2 for retrograde movement of capsids in axons toward the cell body (8). Complete transport of capsids to the nucleus may require both dynein and a virion-packaged kinesin motor (9). Movement of virions and viral components in axons in the anterograde direction during egress depends on kinesin motors, with evidence for Kinesin 1 as especially important for HSV and Kinesin 3 for the related alphaherpesvirus PrV (10–13). Even in epithelial cells, where the distances are much shorter, it is well-established that microtubules and dynein motor function are required for efficient initiation of HSV infection (14–18). However, it is unclear if and how dynein is utilized at the late stages of alphaherpesvirus infection during cytoplasmic assembly and virus egress through the secretory pathway. Studies using the microtubule depolymerizing agent nocodazole have shown it to have only a small inhibitory effect on virus production in epithelial cell lines (19).

We have previously demonstrated the formation of a discrete cytoplasmic virus assembly compartment (cVAC) for HSV-1 in cells of neuronal origin including primary neurons and the neuron-derived cell lines CAD and SH-SY5Y (20). In form and function, it resembles the cVAC formed by HCMV (21–24). Both the HCMV and HSV cVACs are organized in a perinuclear location around the centrosome, suggesting that their components may be gathered to microtubule minus ends by dynein motor transport. Consistent with this idea, the disruption of dynein motor function either by expression of CC1 or depletion of the dynein cargo adapter bicaudal D1 results in cytoplasmic dispersal of tegument proteins ordinarily recruited to the HCMV cVAC (25, 26).

Treatment with nocodazole resulted in the complete disruption of the cVAC in undifferentiated neuronal cells but caused only a small inhibition of virus production, similar to what has been reported in epithelial cells (20). However, the small-molecule dynein ATPase inhibitor, ciliobrevin, resulted in failure to form a neuronal cVAC, suggesting that dynein motor function might be important for cVAC formation and function and for cytoplasmic virus assembly. In this study, we explore this paradox further by determining the role of the dynactin/dynein complex in HSV-1 cVAC formation and cytoplasmic assembly and by exploring the localization of dynein and dynactin in HSV-1 infected cells. We show that HSV-1 infection relocalizes both DIC and dynactin subunit p150Glued from the centrosome to cytoplasmic membranous structures, and the disruption of dynein/dynactin interaction in neuronal cells after viral entry results in cVAC dispersal and a profound cytoplasmic assembly defect. Our results suggest that microtubule motor-mediated transport of assembly components is critical for virus production.

## MATERIALS AND METHODS

### Cell lines

CAD cells (gifts from David Johnson at the Oregon Health and Science University) were maintained in Dulbecco modified Eagle medium (DMEM) F-12 (Thermo-Fisher Scientific) supplemented with 10% fetal bovine serum (FBS) and penicillin-streptomycin (P/S). Vero cells (purchased from ATCC) were maintained in DMEM supplemented with 5% bovine calf serum and penicillin/streptomycin. 293T cells (gifts from Mark Stinski at the University of Iowa) were maintained in DMEM supplemented with 10% FBS and P/S. All cells were cultured in a humidified incubator at 37°C with 5% CO_2_.

### Viruses

HSV-1(F) BAC was a gift from Yasushi Kawaguchi at the University of Tokyo. The HSV-1(F) VP5-null virus was a gift from Prashant Desai at Johns Hopkins University. HSV-1(F) UL35-RFP BAC virus was a gift from Cornel Fraefel at the University of Zürich. The HSV-1(F) pUL51-HA and HSV-1(F) pUL51^Y19A^-HA viruses were described previously (20).

### Plasmids

pEGFP-p150Glued was acquired from Addgene (#36154). All other plasmids generated in this study were assembled using Gibson Assembly Master Mix (New England Biolabs Inc.) according to the manufacturer’s instructions. All PCR primers were purchased from Integrated DNA Technologies. For pcDNA3-pUL51-HA plasmid, the pUL51 fragment was amplified from HSV-1(F) BAC using primers UL51HAfwd and UL51HArev, and backbone fragments were amplified from pcDNA3 with primer pairs vector1fwd + vector1rev and vector2fwd + vector2rev. For the pcDNA3-pUL51^Y19A^-HA plasmid, the mutation was introduced by amplifying pcDNA3-pUL51-HA with the following primer pairs, respectively: Y19A fwd + CDNA8 and CDNA7 + Y19A rev. Fragments for pEGFP-CC1 plasmid were amplified from pEGFP-p150Glued using primers DNCT1, DNCT2, DNCT3, DNCT4, DNCT7, and DNCT8. For 34p-EGFP and 34 p-CC1 plasmids, the 388 bp sequence before the natural start codon of the HSV UL34 gene was amplified from HSV-1(F) BAC using primers pHSV1 + pHSV2. Other fragments for this plasmid were amplified from pEGFP C1 (Promega) or pEGFP-CC1 using primer GFP3, CDNA7, CNDA8, and CDNA9. For the 34p-EGFP-IRES-VP5 plasmid, the internal ribosome entry site (IRES) fragment was amplified from pTUNER-IRES (Promega) with primers IRES10 + IRES11, the VP5 gene was amplified from HSV-1(F) BAC with primer pairs IRES12 + VP5midRev and VP5midFwd + IRES15, the EGFP fragment was amplified from 34p-EGFP with primers pHSV1 + IRES14, and the backbone fragment was amplified from 34p-EGFP with primers CDNA28 + CDNA9. For the 34p-CC1-IRES-VP5 plasmid, the IRES fragment was amplified from pTUNER-IRES (Promega) with primers IRES10 + IRES11, the VP5 gene was amplified from HSV-1(F) BAC with primer pairs IRES12 + VP5midRev and VP5midFwd + IRES13, the CC1 fragment was amplified from 34 p-CC1 with primers pHSV1 + IRES9, and the backbone fragment was amplified from 34 p-CC1 with primers DNCT3 + CDNA9. All primer sequences are listed in Table 1.

**TABLE 1:** Sequences of primers used for plasmid cloning

Primer	Sequence (5'−3')
UL51HAfwd	CCGCCAGTGTGCTGGAATTCATGGCTTCTCTTCTCGGGGCTATATG^*a*^
UL51HArev	CCTCTAGATGCATGCTCGAGTTAAGCGTAATCTGGAACATCGTATGGGTATTG
vector1fwd	CTCGAGCATGCATCTAGAGGGCC
vector1rev	CGCCGCAGCCGAACGACCGAGCGCAGCGAGTCAGTGAGC
vector2fwd	TCGGTCGTTCGGCTGCGG
vector2rev	GAATTCCAGCACACTGGCGGC
GFP3	GTGAGCAAGGGCGAGGAGC
CDNA7	GTTCGGTGTAGGTCGTTCGCTCC
CDNA8	GGAGCGAACGACCTACACCGAAC
CDNA9	CGGAACTCCATATATGGGCTATGAACT
pHSV1	AGTTCATAGCCCATATATGGAGTTCCGCTAGACGCGCGCTACGTCTCG
pHSV2	GCTCCTCGCCCTTGCTCACCATGGCGCCCGCGATG
DNCT1	AAACGCACGGTGTTGGGTCG
DNCT2	AGAATTCGAAGCTTGAGCTCGAGA
DNCT3	GTCGACGGTACCGCGGG
DNCT4	CGACCCAACACCGTGCGTTT
DNCT5	ATGGCACAGAGCAAGAGGCAC
DNCT7	TCTCGAGCTCAAGCTTCGAATTCTCCATCCAAGGAGGAGGAGGG
DNCT8	GGCCCGCGGTACCGTCGACTCACTGCTGTTGCCTCTCCACAGAT
Y19A fwd	GAGGAACAAGCTGAGATGATCCGCGCGGC
Y19A rev	GGATCATCTCAGCTTGTTCCTCGGGGCGCG
IRES9	GGGAGGGAGAGGGGCTCACTGCTGTTGCCTCTCCAC
IRES10	GCCCCTCTCCCTCCC
IRES11	TGTGGCCATATTATCATCGTG
IRES12	CACGATGATAATATGGCCACAATGGCCGCTCCCAACCG
IRES13	CCGCGGTACCGTCGACTTACAGAGCCAGTCCCTTGAGCGG
VP5midFwd	GCAAGCCGAGCTGAATCACC
VP5midRev	GGTGATTCAGCTCGGCTTGC

^*a*^ All primer sequences are shown 5′ to 3′.

### Transfection of cells

For immunofluorescent (IF) microscopy, 1 × 10^5^ cells of CAD, SH-SY5Y, Vero, or HEp2 cells were seeded into 24-well tissue culture plates with 13-mm-diameter coverslips in each well 17–24 h before transfection. CAD and Vero cells were then transfected with X-tremeGENE HP DNA Transfection Reagent (Millipore-Sigma). All transfections followed product protocols.

### Infection and viral growth assay

For all infections, viruses were diluted in the infection medium (DMEM containing 1% heat-inactivated serum and P/S) to achieve a multiplicity of infection (m.o.i.) of 5. Viruses were incubated with cells for 1 h before the removal of the inoculum. Residual viruses from the inoculum were further neutralized with a sodium citrate buffer (50 mM sodium citrate, 4 mM KCl, and adjusted to pH = 3 with HCl), after which cells were washed with the infection medium two times before adding 1 mL of infection medium. For all transfection-superinfections, cells were transfected 24 h before infection. The concentration of infectious virus produced was determined by titration on monolayers of Vero cells by plaque assay. Harvested samples were first frozen, thawed, and then sonicated before titration.

### Immunofluorescence assays

Transfected or infected cells on 13-mm-diameter coverslips were fixed with 3.7% formaldehyde for 20 min before washing with phosphate buffered saline (PBS) two times. The IF buffer used to dilute antibodies or antiserum is a PBS buffer that contains 1% Triton X100, 0.5% sodium deoxycholate, 1% egg albumin, and 0.01% sodium azide. Fixed coverslips were first blocked with IF buffer containing 10% human serum for 30 min before 1 h of primary antibody staining (with 10% human serum) and 1 h of secondary fluorescent antibody staining. The primary antibodies were diluted as follows: rabbit anti-HA epitope tag antibodies (Abcam) 1:500, rabbit anti-UL51 antiserum (gift from Joel Baines at Cornell University) 1:500, rabbit anti-UL11 antiserum (gift from John Wills at Penn State University) 1:1,500, mouse monoclonal anti-gE antibody (Virusys) 1:500, goat anti-p150Glued antibody (Abcam) 1:500, mouse anti-DIC antibody (Santa Cruz Biotechnology) 1:250, mouse anti-GM130 antibody (BD Bioscience), and 1:500 mouse anti-ß-tubulin antibody (Sigma-Aldrich). Secondary antibodies used (Invitrogen) were donkey anti-mouse, anti-rabbit, or anti-goat conjugated with AlexaFluor 488, 568, or 647, and all were diluted 1:1,000. Nuclei were stained with 1:500 DNA dye Hoechst 33342 (Invitrogen). Stained coverslips were mounted with ProLong Diamond Antifade Mountant (Invitrogen) and let dry for 12 h. Confocal images were taken on a Leica DFC7000T confocal microscope equipped with Leica software.

For Pearson co-efficient measurement for protein co-localization, confocal images of 1,024 × 1,024 pixels were captured for random fields. Pearson co-efficient between signals of two different proteins was measured in ImageJ FIJI for each cell. The boundary of each cell was defined by either gE signals or ubiquitous cytoplasmic fluorescent proteins.

For cytoplasmic capsid quantification, the number of cytoplasmic capsids in infected cells was quantified using a modified protocol that has been previously described (27). Hoechst 33342 staining and gE staining were used to define the nuclear and cytoplasmic boundaries of the cell, respectively. ImageJ was used to count the number of red fluorescent protein (RFP) maxima in the cytoplasm.

### Western blotting

For cell lysate samples, cells from each well of a six-well plate were pelleted and lysed in phosphatase inhibitor radioimmunoprecipitation buffer (P.I. RIPA) buffer (50 mM Tris pH = 7.5, 150 mM NaCl, 5 mM NaF, 5 mM sodium vanadate, 1 mM EDTA, and 1% triton X-100). Lysates were then sonicated for 10 s before adding loading dye (5× stocks contain 4% SDS, 0.25M Tris buffer pH = 6.8, 0.6 mg/mL bromphenol blue, and 30% glycerol) and 2-mercaptoethanol (to a final concentration of 1%). Proteins were denatured by incubating at 85°C for 8 min. Denatured samples were then separated on SDS-PAGE by size and transferred to nitrocellulose membranes before blocking in 20 mM Tris pH 7.5, 100 mM NaCl, 5% nonfat milk, and 0.2% Tween 20 for at least 1 h. Then, membranes were probed with either 1:5,000 rabbit anti-EGFP antiserum (gift from Craig Ellermeier at the University of Iowa), 1:500 mouse anti-ICP27 monoclonal antibody (Virusys), 1:1,000 rabbit anti-gE antiserum (gift of H. Friedman), or 1:1,000 rabbit anti-actin antiserum and detected by alkaline phosphatase-conjugated secondary antibodies diluted 1:1,000 and obtained from Millipore/SIGMA (anti-rabbit) or Invitrogen (anti-mouse).

### Statistical analysis

All statistical analyses were conducted by using Student’s *t* test, one-way ANOVA, or one-way repeated measures ANOVA in GraphPad Prism 8. The number of cytoplasmic capsid local maxima and plaque-forming unit (PFU) number in infectious particle titration were log-transformed before statistical analyses.

## RESULTS

### Association of dynein and dynactin with the HSV-1 cVAC

We have shown that HSV forms a cVAC around the centrosome adjacent to the nucleus in several neuronal cells, including primary mouse cerebral neurons and in the neuronal cell lines CathA (CAD) and SH-SY5Y (20). We have further shown that the formation of this cVAC is sensitive to the microtubule depolymerizing drug nocodazole and therefore dependent on an intact microtubule network (20). The centrosomal position of the cVAC and the dependence on the microtubule network suggested that the substrates of assembly, including nucleocapsids and vesicles containing integral membrane and membrane-associated virus structural proteins, might be gathered at this site by minus end-directed transport on microtubules using a dynein motor. Consistent with this, the small molecule dynein ATPase inhibitor ciliobrevin also prevented cVAC formation (20). Since the specificity of ciliobrevin is uncertain (28), we further explored the function of dynein in cVAC formation and HSV-1 assembly in CAD cells.

**Fig 1:**
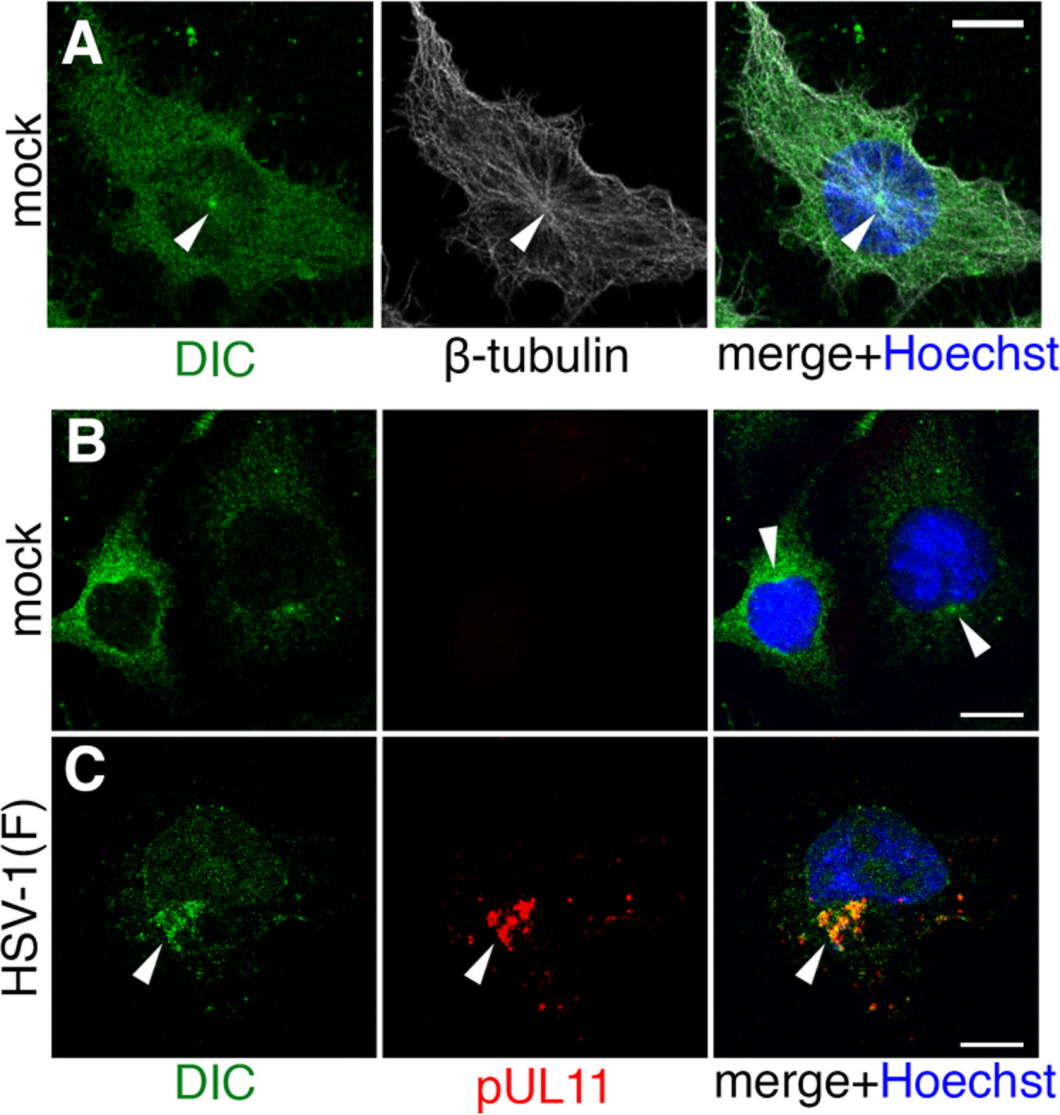
Localization of DIC in infected CAD cells. (**A and B**) Mock-infected CAD cells that were immunofluorescently stained for indicated proteins. The arrowhead indicates the position of the centrosome. (**C**) CAD cells infected with HSV (at m.o.i. = 5 for 12 h before fixation and staining). The arrowhead indicates the position of the cVAC. All scale bars represent 10 µm.

The hypothesis that dynein promotes the formation and maintenance of the cVAC predicts that some dynein should concentrate at the cVAC. To test this, we determined the localization of DIC in CAD cells that were uninfected or that had been infected for 12 h with HSV-1 (Fig. 1). As expected, in uninfected cells, dynein was present throughout the cytoplasm and concentrated at the perinuclear centrosome (situated atop the nucleus in the view shown; Fig. 1A). Infection with HSV-1 changed the localization of DIC such that it was depleted from most of the cytoplasm and concentrated at a perinuclear accumulation of vesicular structures (Fig. 1B and C). This cluster of vesicles corresponds to the cVAC, as shown by co-localization with the viral membrane-associated tegument protein pUL11, which we have previously shown to be cVAC associated in infected neuronal cells (20). Thus, dynein accumulates at the cVAC in infected CAD cells.

**Fig 2:**
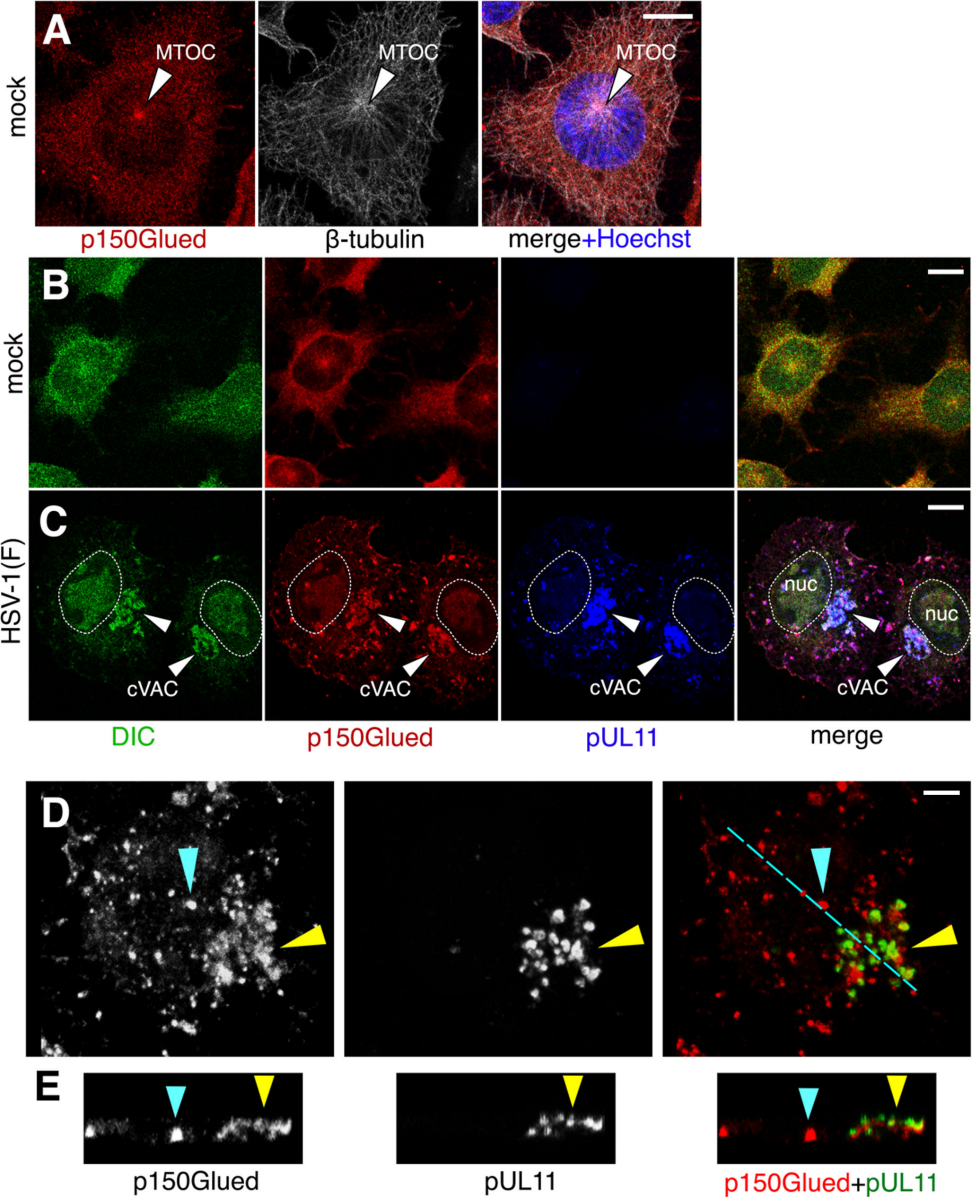
Localization of DIC and p150Glued in infected CAD cells. (**A and B**) Digital confocal images of mock-infected CAD cells that were immunofluorescently stained for indicated proteins are shown. The arrowhead in (**A**) indicates the MTOC. (**C–E**) CAD cells infected with HSV at m.o.i. = 5 for 12 h before fixation and staining are shown. In (**C**), arrowheads indicate the position of the cVAC, where pUL11 and p150Glued co-localize. The white dotted outline indicates the boundary of the nucleus defined by Hoechst staining. In (**D and E**), cyan and yellow arrowheads indicate landmark p150Glued puncta outside and inside the cVAC, respectively. The cyan line in (**D**) indicates the line along which the Z-sections shown in (**E**) were taken. Scale bars in A–C represent 10 µm. Scale bar for D and E represents 4 µm.

**Fig 3:**
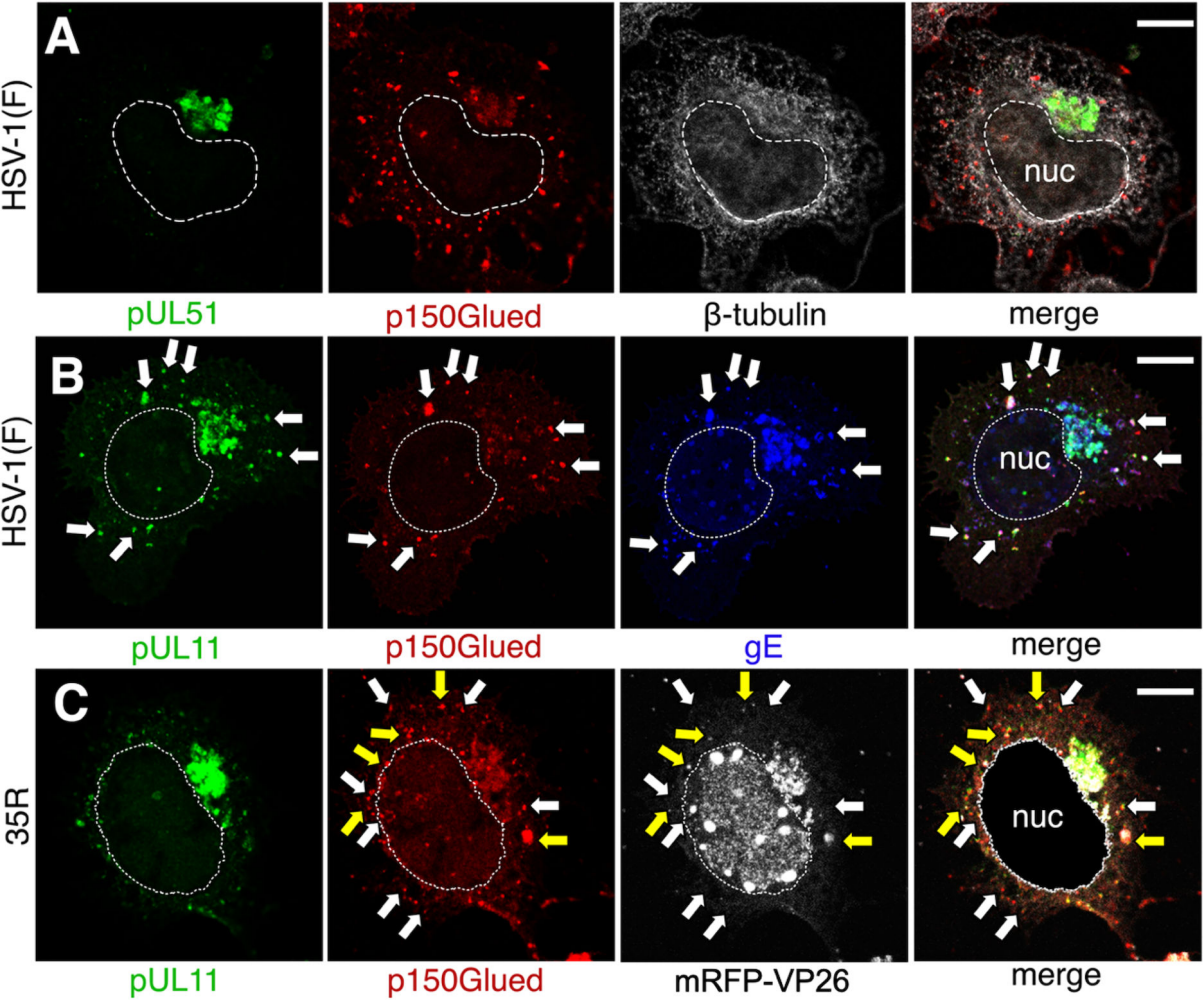
Localization of viral structural components relative to p150Glued. Digital images of CAD cells infected at m.o.i. = 5 for 12 h are shown. (**A–C**) CAD cells infected with HSV(F) (**A and B**) or HSV-1 35R (**C**). In (**B**), arrows indicate puncta outside the cVAC, where pUL11, p150Glued, and gE co-localize. In (**C**), white arrows indicate p150Glued puncta outside the cVAC that did not contain detectable capsid signal, while yellow arrows indicate p150Glued puncta outside the cVAC that co-localizes with capsids. All scale bars represent 10 µm.

Dynein motor function is dependent on the association of the dynein motor with dynactin (5, 6). To determine whether active dynein might be present at the cVAC, we also observed the localization of dynactin in infected and uninfected CAD cells (Fig. 2 and 3). As expected, the localization of p150Glued in uninfected cells was very similar to that of DIC (Fig. 2A). In infected cells, p150Glued also concentrated at the cVAC, suggesting that active dynein motors were present there (Fig. 2B and C, Fig. 3A through C), although the degree of concentration was variable (compare dynactin distributions in Fig. 3A through C). Interestingly, within the cVAC, the association of p150Glued with pUL11-containing membrane puncta appeared to be tangential. That is, the cVAC membranes marked by pUL11 abutted filaments or sheets that contained p150Glued (Fig. 2D and E, yellow arrowheads).

### Recruitment of gE, but not pUL51, to the cVAC by retrieval from the plasma membrane

Another interesting feature of p150Glued localization in infected cells was that, in addition to its localization at the cVAC, it was also found associated with two populations of cytoplasmic vesicles outside of the cVAC. Some of these co-localized with gE, but not pUL51 (Fig. 3B), and some contained both pUL11 and fluorescently labeled capsids (Fig. 3C).

**Fig 4:**
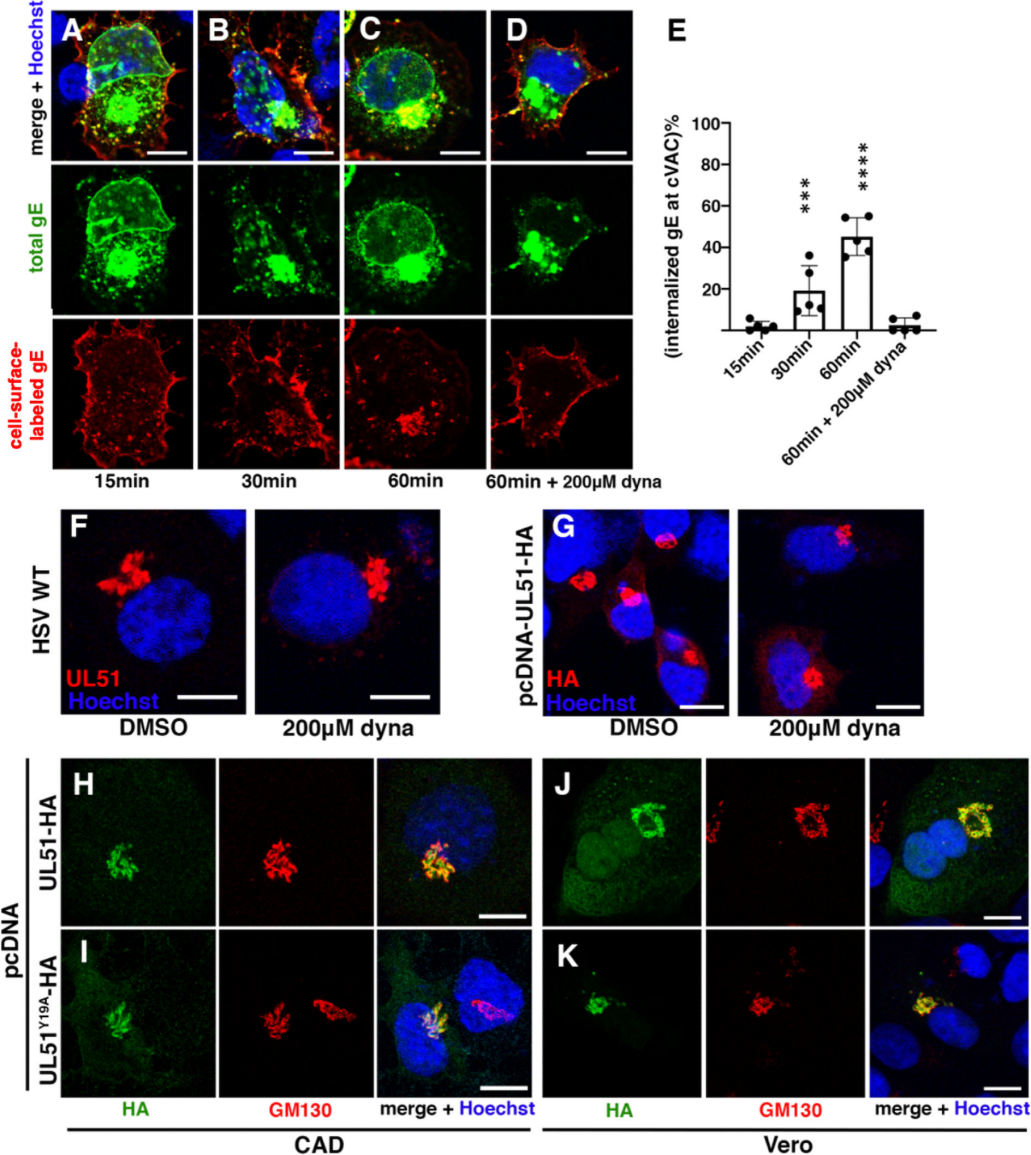
Retrieval of gE but not pUL51 from the plasma membrane. (**A–C**) CAD cells were infected with wild type (WT) HSV-1(F) BAC virus at m.o.i. = 5 for 11 h before replacing media with new media containing 1:1,000 mouse anti-gE antibody (internalized gE; red) and incubated for the indicated time. After incubation, cells were then fixed, permeabilized, and stained with rabbit anti-gE antibody (total gE; Green). (**D**) Cells were treated as in (**C**), except the new medium also contained 200 µM dynasore. (**E**) Quantification of internalized gE (red) in (**A–D**) that concentrated to the cVAC, which was defined by concentrated total gE (green) at one side of the nucleus. Each dot represents an individual experiment, with 45–80 cells counted. Statistical comparisons used one-way ANOVA with samples compared to the 15-min time point. Error bars = SD. ***=*P* < 0.001 and ****=*P* < 0.0001. (**F**) CAD cells were infected with WT HSV-1(F) BAC virus for 12 h before treating with 200 µM dynasore for 1 h. (**G**) CAD cells were transfected with the indicated plasmid for 48 h before being treated with 200 µM dynasore for 1 h. (**H–K**) CAD (**H and I**) or Vero (**J and K**) cells were transfected with indicated plasmids 48 h before fixation, permeabilization, and immunostaining. All scale bars = 10 µm.

The distinction between viral structural proteins that do not co-localize with p150Glued outside the cVAC (e.g., pUL51) and those that do (pUL11 and gE) might reflect different mechanisms of recruitment to the cVAC. Retrograde transport toward the centrosome would be expected for proteins that are retrieved from the plasma membrane. Plasma membrane retrieval of herpes simplex envelope glycoproteins in epithelial cells is well documented (29–31). The HSV-1 gE cytoplasmic tail contains two YXXΦ motifs that might mediate retrieval from the plasma membrane, and retrieval of its varicella zoster virus (VZV) homolog from the plasma membrane dependent on a similar motif has been demonstrated (32–34). To determine whether gE is also retrieved from the plasma membrane in neuronal cells, CAD cells were infected with HSV-1(F) for 11 h, and then cell surface gE was labeled by incubation with anti-gE monoclonal antibody. At various times after antibody addition, cells were fixed, permeabilized, and stained using an anti-gE rabbit antiserum to detect total gE. Between 15 and 60 min after antibody addition, cell-surface-labeled gE diminished at the plasma membrane and accumulated at the perinuclear cVAC (Fig. 4A, B, C and E). This internalization was inhibited by incubation of the cells with the endocytosis inhibitor, dynasore (Fig. 4D and E), showing that at least some of the gE that arrives at the cVAC does so by endocytic retrieval and retrograde transport from the cell surface. Note that the signal for total gE in this experiment is dominated by gE that had accumulated at the cVAC in the 11 h preceding the beginning of surface labeling. How much of this arrived by retrieval from the plasma membrane is not clear.

pUL51 also contains a YXXΦ motif near the N-terminus that is well conserved among its homologs in the herpesviruses (35). This motif has been shown to mediate retrieval of its HCMV homolog, pUL71, from the plasma membrane (36). pUL51 is a palmitoylated, membrane-associated protein whose sequences are completely contained within the cytoplasm, so it cannot be surface-labeled like gE (37). However, in CAD cells infected with wild-type HSV-1, pUL51 was highly concentrated at the perinuclear cVAC and was not detectable at the plasma membrane (Fig. 4F). Moreover, this localization pattern did not change in the presence of dynasore, suggesting that the pUL51 absence at the cell surface is not simply due to extremely efficient retrieval. The same was true when pUL51 was expressed in the absence of other viral proteins (Fig. 4G). Mutation of the tyrosine of the N-terminus proximal YXXΦ motif on HCMV pUL71 results in its accumulation at the plasma membrane (36). The same is not true for HSV-1 pUL51 since both wild-type and a Y19A mutant were tightly localized to the Golgi in either CAD or Vero cells as shown by co-localization with the cis-Golgi marker, GM130 (Fig. 4H through K).

### Dynactin function is essential for the production of HSV infectivity in CAD cells

**Fig 5:**
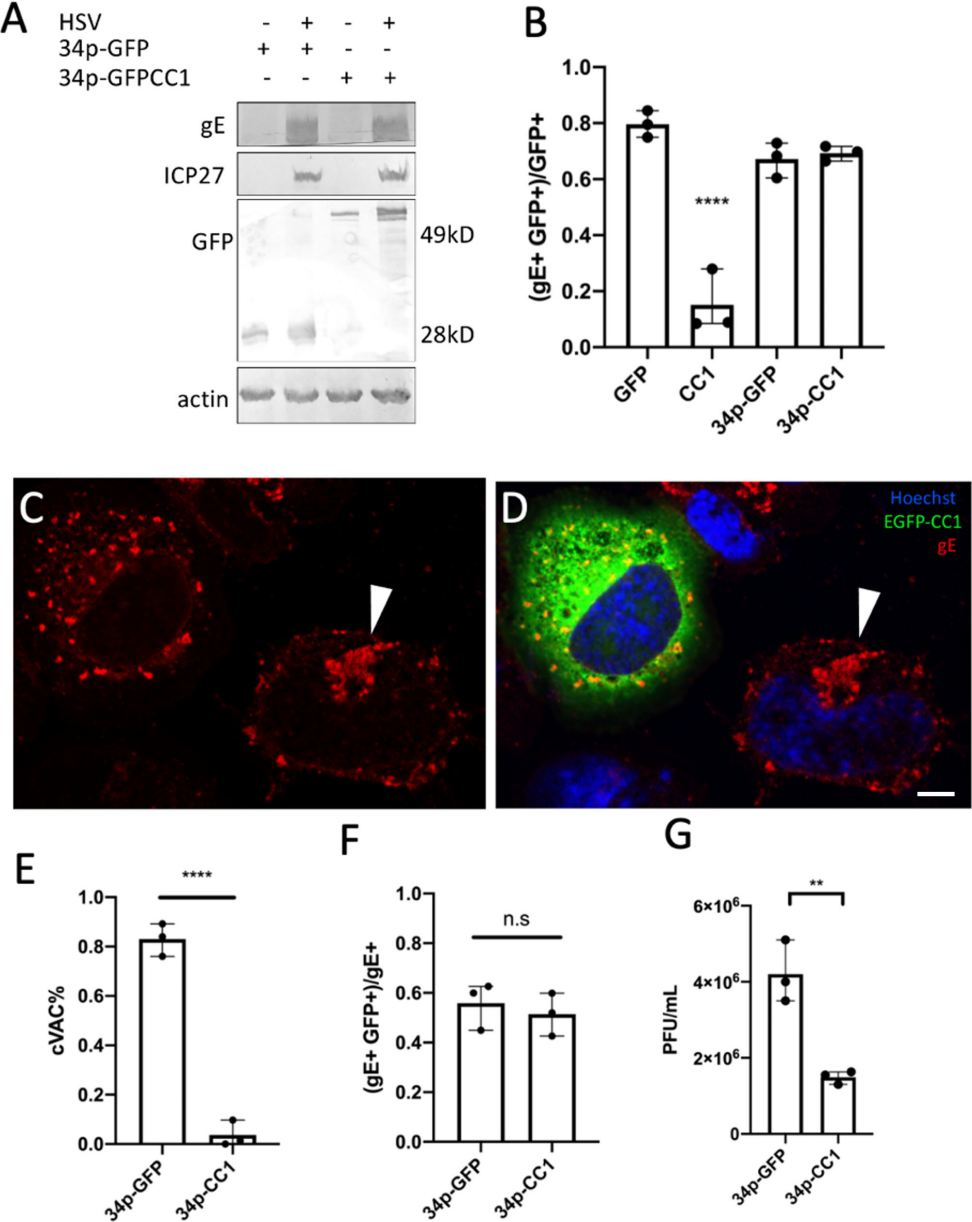
Disrupting dynein function by CC1 overexpression impedes viral production at a post-entry phase. (**A**) Immunoblots showing HSV-1 infection-induced, UL34 promoter-controlled expression of EGFP from plasmid 34p-GFP, or CC1 from plasmid 34 p-CC1 in CAD cells. (**B**) Efficiency of infection of cells transfected with plasmids expressing EGFP or CC1 from either the constitutive HCMV promoter (left two bars) or from the UL34 promoter (right two bars). The Y axis shows the fraction of total transfected cells that expressed both the transgene and the viral gE. (**C and D**) Confocal images of 34p-CC1-transfected, HSV-1-infected CAD cells. Nuclei are stained with Hoechst33342 in blue; CC1-EGFP is shown in green, and the location of glycoprotein gE is shown in red. The white arrowhead indicates the cVAC in the un-transfected cell. (**C**) Shows only gE stain. (**D**) Shows a merged image. The scale bar represents 10 µm. (**E**) Fraction of EGFP or CC1 and gE double-positive CAD cells that form a cVAC in confocal images obtained 12 h post-infection (h.p.i.). At least 50 cells were imaged and scored for the presence of a cVAC in three independent experiments. (**F**) Fraction of infected CAD cells that express EGFP or CC1 in confocal images obtained 12 h.p.i. At least 104 infected (i.e., gE-expressing cells) were imaged at 12 h.p.i. and scored for expression of EGFP or CC1 in three independent experiments. (**G**) Viral production in transfected-superinfected CAD cells at 16 h after infection at m.o.i. = 5. In panel B, one-way ANOVA was used to compare to the GFP transfected condition. In panels E–G, a two-tailed Student’s *t* test was used for analysis. In panels (**B and E–G**), **=*P* < 0.01, ***=*P* < 0.001, ****=*P* < 0.0001, and data represent mean ± range.

Overexpression of the p150Glued CC1 domain-EGFP fusion protein has been shown to disrupt dynein motor function by interfering with dynactin/dynein complex formation (7, 38). To confirm that dynein motor function was required for cVAC formation, we wanted to specifically disrupt that function by transfecting cells with plasmids that expressed either EGFP or EGFP fused to the CC1 domain of p150Glued (hereon referred to as CC1) and then superinfecting with wild-type HSV-1. It has previously been shown, however, that initiation of infection by HSV is facilitated by dynein, so the expression of a dynactin inhibitor prior to infection might affect both early and late phases of HSV replication (39). To limit the effect of CC1 overexpression to the late phase of infection, CC1 or EGFP was expressed under the control of the HSV UL34 promoter, which has been shown to mediate infection-inducible expression (40). To test this system, we transfected cells with constructs in which EGFP or CC1 was expressed either using the HCMV promoter or the UL34 promoter, superinfected with wild-type HSV-1(F), and then assayed for protein expression (Fig. 5A) and efficiency of infection (Fig. 5B). As expected, we observed infection-inducible expression of EGFP and CC1 (Fig. 5A) but with some leaky expression of the UL34 promoter-driven CC1 in uninfected cells. We investigated whether this leaky expression impedes the establishment of viral infection by quantifying the fraction of different populations of cells in transfected-superinfected cell cultures by confocal imaging (Fig. 5B). Expression of CC1 from the constitutive HCMV promoter diminished the fraction of cells that became infected by roughly fivefold, consistent with an important role for dynactin in initiation of infection of CAD cells. In contrast, expression of CC1 from the UL34 promoter did not inhibit initiation of infection or events up to and including late gene expression since there was no difference in the frequency of cells that express gE between cells that expressed EGFP and those that expressed CC1.

As shown in Fig. 5C through E, a single cVAC formed in most infected cells that expressed no CC1, whereas cVAC formation was almost completely inhibited in infected cells that expressed CC1 from the UL34 promoter, and gE was dispersed throughout the cytoplasm in small puncta. This showed that the formation of the cVAC depends on dynein motor function at a point after initiation of infection. To determine the effect of infection-inducible CC1 expression on virus production, parallel cultures of CAD cells were transfected for 24 h with plasmids that expressed infection-inducible EGFP or CC1, infected for an additional 16 h with HSV-1, and in one set of cultures, the frequency of infected, transfected cells was determined by confocal microscopy (Fig. 5F), and in the other, total infectious virus produced in the culture (i.e., cell associated and released virus) was measured by plaque assay (Fig. 5G). About half of the infected cells expressed CC1 or EGFP in these experiments, and expression of CC1 from the UL34 promoter diminished virus production roughly twofold. This result suggested that dynein disruption might severely inhibit viral replication.

**Fig 6:**
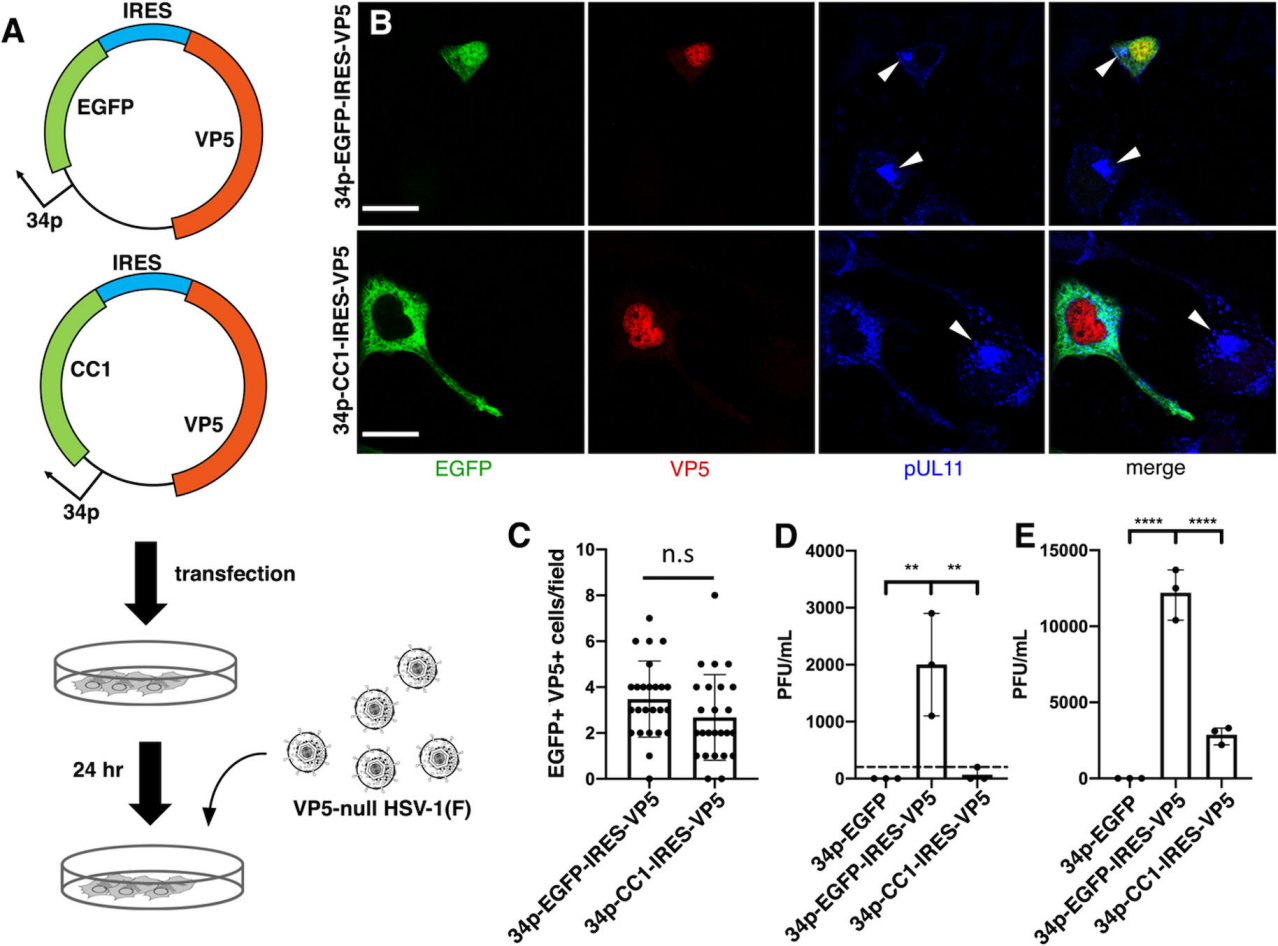
Inhibition of dynactin function severely impairs virus production. (**A**) Schematic diagram of dual expression plasmids and experimental design. (**B**) Confocal images of CAD cells transfected with indicated plasmid before infection with VP5-null virus for 12 h. White arrowheads indicate the position of cVACs observed in cells that do not express CC1. (**C**) The number of EGFP-VP5 double-positive cells was counted for at least 25 random fields. (**D and E**) Viral production in CAD (**D**) or Vero (**E**) cells after transfection for 24 h with indicated plasmid and then infection for an additional 18 h with VP5-null virus at m.o.i. = 5. Progeny virion production was quantified by plaque assay on VP5-complementing cells. In panel C, a two-tailed Student’s *t* test was used for analysis. In panels D and E, a one-way ANOVA was performed on log-transformed data using Tukey’s multiple comparisons test. For all panels, **=*P* < 0.01, ***=*P* < 0.001, ****=*P* < 0.0001, and data represent mean ± range. The dotted line in (**D**) indicates the limit of detection.

To define the magnitude of growth defect caused by dynein disruption, we constructed infection-inducible plasmids that not only express EGFP or CC1 but also the major capsid protein, VP5 (Fig. 6A). CAD and Vero cells were transfected with these plasmids and then infected with VP5-null HSV, which should only produce progeny virions in EGFP- or CC1-overexpressing cells that also trans-complement VP5. Cell cultures that were transfected with these plasmids and then infected with VP5-null virus expressed both EGFP or CC1 and VP5 (Fig. 6B). The frequency of transfected cells was the same for both the EGFP- and CC1-expressing plasmids (Fig. 6C). The defect in the production of total infectivity due to CC1 expression was greater than 200-fold in CAD cells showing that active dynein is critical for late events in HSV replication (Fig. 6D). Interestingly, inhibition of infectivity production was only about fivefold in Vero cells (Fig. 6E).

### Dynactin inhibition affects a step after capsid nuclear egress

**Fig 7:**
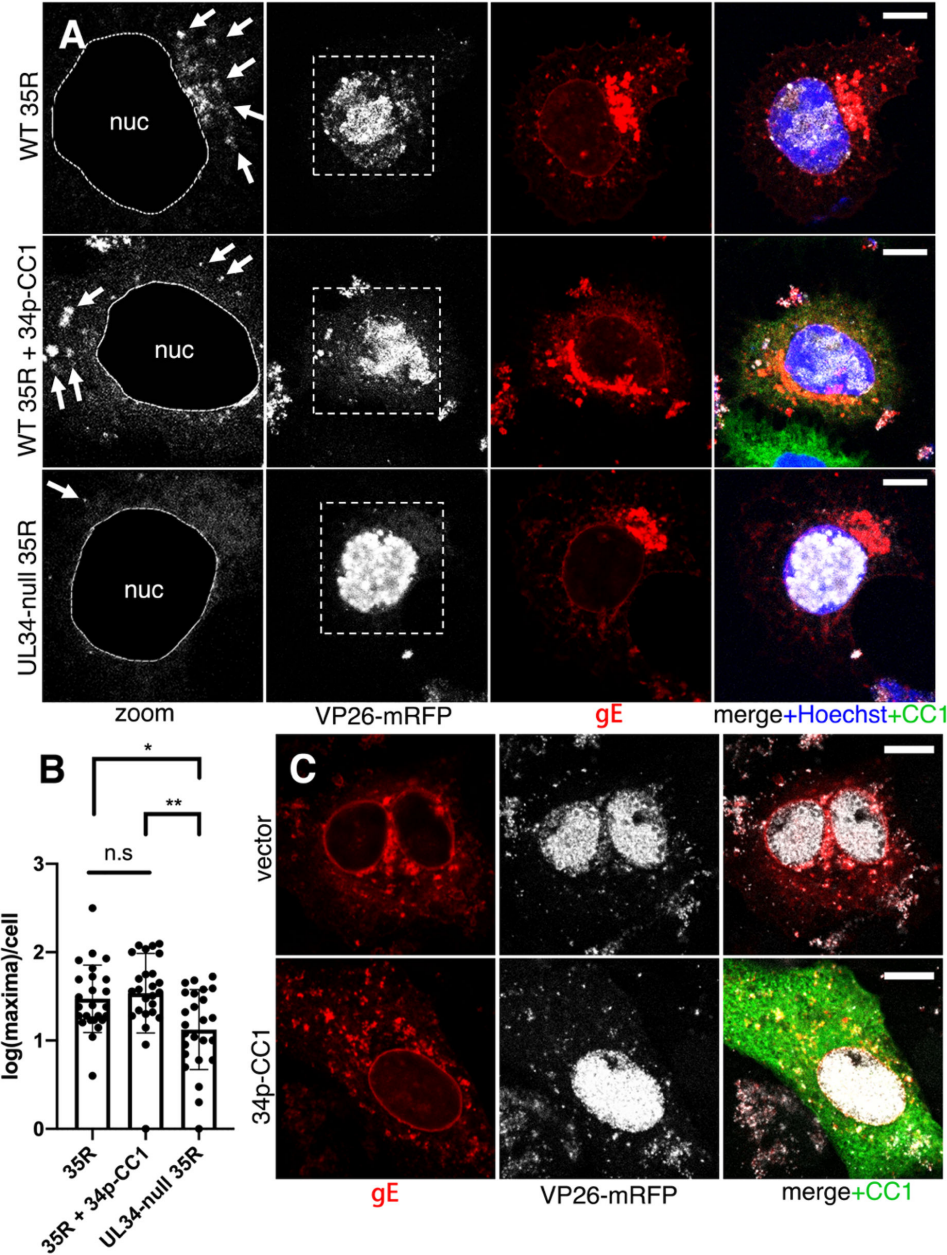
CC1 overexpression does not prevent capsid nuclear egress. (**A**) Confocal images of CAD cells infected with HSV-1 35R virus, which expresses an monomeric red fluorescent protein (mRFP) fusion to the capsid protein VP26. Nuclei are stained with Hoechst33342 in blue; CC1-EGFP is shown in green, gE detected with anti-gE antibody is shown in red, and mRFP-VP26 is shown in white. In the middle row (CC1 expressing cells), CAD cells were transfected and superinfected as described in Fig. 5. In zoomed images in the left-most column, nuclei are masked (guided by Hoechst staining). White arrows indicate individual or aggregated cytoplasmic capsids. (**B**) Quantification of cytoplasmic capsids per cell by counting local mRFP maxima in the cytoplasm of 25 cells per condition. (**C**) Same as (**A**) but in Vero cells. In panel B, a one-way ANOVA was performed using Tukey’s multiple comparisons test. All scale bars represent 10 µm. *=*P* < 0.05 and **=*P* < 0.01. Data represent mean ± SD.

The most likely site of inhibition of virus production is cytoplasmic assembly, but it was possible, in principle, that inhibition of earlier events, including DNA replication, late gene expression, or nuclear egress of capsids, might affect virus production. Normal expression of the true-late gE protein indicates that DNA replication and late gene expression are unaffected (Fig. 5B). To determine whether CC1 expression inhibits events that preceded arrival of capsids in the cytoplasm, we quantified cytoplasmic capsids in cells infected with a recombinant virus that expresses a fluorescently tagged capsid protein as previously described (Fig. 7A) (27). In CAD cells that overexpressed CC1, the number of cytoplasmic capsids was no lower than in infected cells that did not express CC1 and was significantly higher than in cells infected with a UL34-null virus, which is defective in nuclear egress (Fig. 7B) (41). We also did not observe inhibition of capsid nuclear egress in CC1-overexpressing Vero cells (Fig. 7C). From these data, we conclude that CC1 overexpression specifically interferes with cytoplasmic assembly events.

## DISCUSSION

The size of herpesvirus capsids and envelope glycoprotein-containing vesicles and the viscosity of the cytoplasm strongly suggest that microtubule-dependent trafficking should be important for HSV cytoplasmic assembly and egress. There is also evidence that viral envelope glycoproteins are delivered to assembly sites after retrieval from the plasma membrane, and secondary envelopment may take place at membranes derived from the plasma membrane by endocytosis (42). Since retrograde transport of endocytic vesicles depends on microtubule-associated dynein motors (43), it is reasonable to hypothesize that microtubule-dependent dynein-mediated trafficking might be essential for HSV secondary envelopment. Paradoxically, nocodazole treatment of epithelial cell lines, including Vero, Hep-2, and 413(TK+) has been shown to cause only small defects in virion production and no defect in virus release to the medium (19). Virus assembly in epithelial cell types occurs at peripheral, dispersed sites, and how this might affect the requirement for vesicular and capsid transport is unclear. We previously showed that HSV forms a cVAC around the centrosome in mouse neuronal CAD cells, and its formation depends on intact microtubule networks (20). Similar to epithelial cells, however, disruption of microtubule filaments by nocodazole did not result in a substantial viral growth defect. Here, we have shown that dynein and dynactin localize to the cVAC, that recruitment of at least one of the HSV glycoproteins to the cVAC is dependent on plasma membrane retrieval, and that cVAC formation and cytoplasmic virus assembly are strongly dependent on dynein motor function.

In both infected and uninfected CAD cells, dynein and dynactin were concentrated around the MTOC, but the appearance of both MTOC-associated and dispersed cytoplasmic proteins differed. In uninfected CAD cells, MTOC-concentrated dynein and dynactin appeared as fine puncta. In contrast, in infected cells, the cVAC/MTOC-associated dynein and dynactin adopted the distribution of the vesicles that contained the viral structural proteins and formed sheets associated with those vesicles (Fig. 2). The dynein and dynactin that were not concentrated to the MTOC/cVAC in infected cells also formed large puncta that, in most cases, co-localized with virus structural proteins. These results suggest that dynactin and dynein are recruited from the cytoplasm to HSV protein-containing vesicles both at the cVAC and in the more peripheral cytoplasm. The redistribution of dynein and dynactin from a tightly centered MTOC in uninfected cells to a broader distribution within the cVAC in infected cells suggests the possibility that those membranes may also serve as MTOCs during infection of CAD cells. Many previous studies have shown that Golgi membranes can serve as non-centrosomal MTOCs (44–46), and studies in primary human fibroblasts have shown that MTOCs form late in infection in association with dispersed Golgi membranes (47), suggesting that this may be a common feature of HSV infection whether or not the assembly apparatus is dispersed or concentrated in a cVAC. Furthermore, Golgi-derived membranes in the cVAC have been shown to be an MTOC in HCMV-infected cells (48), suggesting that Golgi-associated MTOC function may be a conserved feature of herpesvirus assembly mechanisms.

HSV envelope glycoproteins are retrieved from the cell surface to dispersed, peripheral assembly sites in epithelial cells (31). Not needing retrograde transport to the perinuclear centrosome might explain the nocodazole resistance in these cell types. We determined whether viral membrane-associated structural proteins might also be recruited to the neuronal assembly site by retrieval. We determined that, like envelope glycoproteins in epithelial cells and like its VZV homolog, HSV-1 gE is also retrieved from the plasma membrane and is transported to the cVAC. This suggests that microtubule- and dynein-dependent retrograde trafficking must be important for cVAC formation. Since pUL11 forms a stable complex with gE (49, 50), this retrograde trafficking may partly account for the co-localization of pUL11 and gE with dynactin outside of the cVAC. Surprisingly, we also observed that, unlike its HCMV homolog, HSV-1 pUL51 is not retrieved from the plasma membrane since it does not accumulate at the plasma membrane when endocytosis is inhibited with dynasore or when its YXXΦ putative internalization motif is altered by mutation. We also did not observe co-localization of pUL51 with dynactin outside of the cVAC. We conclude that viral membrane-associated proteins arrive at the cVAC in CAD cells by different routes. They must, nonetheless, be incorporated into the same membrane for the formation of a virus particle that contains the full complement of proteins. How this is achieved is as yet unclear.

Dynein motor function has previously been shown to be important for the initiation of HSV-1 infection (14–18), and our results confirm this since transfection with a dominant negative inhibitor of dynein/dynactin interaction (CC1) 24 h prior to infection diminished the number of cells that expressed the viral protein gE. In contrast, expression of CC1 from a virus-inducible promoter did not inhibit the initiation of infection but strongly inhibited both cVAC formation in CAD cells and production of infectivity in both CAD and Vero cells. The co-localization of dynein and dynactin at the cVAC, coupled with the strong effect of CC1 expression on cVAC organization and virus assembly, shows that active dynein motors are present at the cVAC and critical for its structure and assembly function. Because we measured total culture infectivity, we do not know whether dynein function might also be important for virus trafficking to the cell surface. The defect in virus production occurred at a step following capsid nuclear egress, indicating that it was due specifically to inhibition of cytoplasmic virus assembly. The degree of inhibition was substantially greater in CAD (~1,000 fold) than in Vero cells (about fivefold), and this might reflect the greater proximity of dispersed Vero assembly sites to the plasma membrane.

The combination of nocodazole resistance for virus production in neuronal cells, combined with profound sensitivity to inhibition of dynein motor function, suggests that concentrations of nocodazole that are sufficient to eliminate visible microtubule networks and that can disrupt cVAC organization must, nonetheless, leave a nocodazole-resistant functional network for dynein-mediated transport. In primary human fibroblasts, HSV-1 was shown to promote the formation of stabilized, acetylated microtubules late in infection in a manner dependent on phosphorylation of GSK3β by the viral protein kinase, pUS3 (47). Also, it has previously been shown that microtubules are modified by acetylation late in HSV-1 infection of Vero cells, and ectopic expression of the viral tegument protein VP22 can stabilize microtubules and make them nocodazole resistant (51). VP22 is not critical for assembly in Vero cells, and the significance of this activity is, therefore, unclear (52, 53). Whether either or both of these mechanisms are responsible for the strong dynactin dependence of virus assembly in cells with a centralized assembly apparatus is an interesting question for further study.

## Data Availability

Data presented here, including raw images used for preparation of composite figures and data tables used for preparation of graphs, are available from the corresponding author upon request.
